# CX-5461 induces radiosensitization through modification of the DNA damage response and not inhibition of RNA polymerase I

**DOI:** 10.1038/s41598-022-07928-4

**Published:** 2022-03-08

**Authors:** Stacey L. Lehman, Kayla R. Schwartz, Shrankhla Maheshwari, Kevin Camphausen, Philip J. Tofilon

**Affiliations:** Radiation Oncology Branch, National Cancer Institute, 10 Center Drive-MSC 1002, Building 10, B3B69B, Bethesda, MD 20892 USA

**Keywords:** Cancer, Oncology

## Abstract

Increased ribosome biogenesis is a distinguishing feature of cancer cells, and small molecule inhibitors of ribosome biogenesis are currently in clinical trials as single agent therapy. It has been previously shown that inhibiting ribosome biogenesis through the inhibition of nuclear export of ribosomal subunits sensitizes tumor cells to radiotherapy. In this study, the radiosensitizing potential of CX-5461, a small molecule inhibitor of RNA polymerase I, was tested. Radiosensitization was measured by clonogenic survival assay in a panel of four tumor cell lines derived from three different tumor types commonly treated with radiation. 50 nM CX-5461 radiosensitized PANC-1, U251, HeLa, and PSN1 cells with dose enhancement factors in the range of 1.2–1.3. However, 50 nM CX-5461 was not sufficient to inhibit 45S transcription alone or in combination with radiation. The mechanism of cell death with the combination of CX-5461 and radiation occurred through mitotic catastrophe and not apoptosis. CX-5461 inhibited the repair and/or enhanced the initial levels of radiation-induced DNA double strand breaks. Understanding the mechanism of CX-5461-induced radiosensitization should be of value in the potential application of the CX-5461/radiotherapy combination in cancer treatment.

## Introduction

A defining characteristic of tumor cells is an increase in nucleolar size and number, which reflects an increase in ribosome biogenesis and the elevated level of protein synthesis needed to maintain tumor cell proliferation^1^. More recent studies have also suggested that the relationship between the hyperactivation of ribosome biogenesis and tumorigenesis involves not only an increase in global protein synthesis, but also an alteration in the pattern of translated mRNAs^2^. Typically, many of the mRNAs encoding oncoproteins, growth factors, survival factors, and cell cycle regulators have a low affinity for the translational machinery and are outcompeted by mRNAs with high affinity, i.e., housekeeping genes, when the number of ribosomes is limiting^2^. However, an increase in ribosome biogenesis reduces this limiting factor, facilitating the translation of mRNAs whose protein products participate in tumor initiation and progression. Consistent with this process, the upregulation of various steps of ribosome biogenesis have been causally linked to the reprograming of translational control in tumor cells^3^. Thus, to simultaneously target the elevated protein synthesis and altered translational control in tumor cells, inhibiting ribosome biogenesis has been suggested as a cancer treatment strategy^1,4^.

Ribosome biogenesis may also provide a target for tumor cell radiosensitization. This is based on our previous work showing that the inhibition of rRNA nuclear export selectively enhances tumor cell radiosensitivity^5^. Specifically, Selinexor, an inhibitor of the nuclear transport receptor exportin 1 (XPO1), was found to inhibit the nuclear export of rRNA, resulting in a decrease in ribosome biogenesis along with the expected reductions in translational efficiency and protein synthesis. This effect was found in tumor cells, but not normal cells, which was accompanied by an increase in tumor cell radiosensitivity. The tumor selectivity of the Selinexor-mediated radiosensitization could, at least in part, be attributed to the overexpression of XPO1 in tumor cells. An alternative approach to investigating the role of ribosome biogenesis as a determinant of radiosensitivity is to target RNA polymerase I (Pol I), an essential molecule in the transcription of rDNA. Pol I mediates the production of the 45S rRNA precursor, which is modified and processed to form mature 18S, 5.8S and 28S rRNAs. The rRNAs are then assembled with ribosomal proteins and exported into the cytoplasm^6^. Several Pol I inhibitors have been identified, with CX-5461 as one of the first to proceed to clinical trials as a single modality cancer treatment^7–9^. CX-5461 disrupts the binding of the SL1 pre-initiation complex and Pol I to rDNA promotors, reducing rDNA transcription and the synthesis of 45S rRNA precursors^8^. Studies using mouse models of hematologic and solid cancers showed that anti-tumor effects of CX-5461 corresponded to an inhibition of Pol I^4,10–13^, providing the basis for clinical trials.

The combination of CX-5461 and radiation was initially reported to result in a synergistic increase in tumor cell death^14^. However, in that study the evaluation of tumor cell death was limited to measuring cell proliferation at 4 days after irradiation, which does not provide an accurate measure of radiosensitivity^15^. Moreover, in that study Pol I activity was determined only at CX-5461 doses significantly greater than those necessary for the maximum synergy with radiation^14^. In the study described here, we determined the effects of CX-5461 on the radiosensitivity of a panel of human tumor cell lines to further investigate ribosome biogenesis as a target for radiosensitization. In each of the cell lines, exposure to CX-5461 resulted in a significant increase in radiation-induced cell death as measured by clonogenic survival. However, at a drug concentration sufficient for radiosensitization, ribosome biogenesis was not affected. Of note, although most studies have focused on Pol I inhibition, CX-5461 has also been shown to bind to and stabilize G-quadruplex (G4) DNA, which may influence the DNA damage response after irradiation^16^. Consistent with this interaction, CX-5461 at the radiosensitizing dose modified the induction and/or repair of radiation-induced DNA double strand breaks. Our data indicate that while CX-5461 induces radiosensitization, the mechanism involves the DNA damage response rather than a reduction in ribosome biogenesis.

## Materials and methods

### Cell lines and treatments

PANC-1 (pancreatic adenocarcinoma), HeLa (cervical carcinoma), and PSN1 (pancreatic adenocarcinoma) cell lines were obtained from American Type Culture Collection (ATCC). The U251 (glioblastoma) cell line was obtained from the Division of Cancer Treatment and Diagnosis Tumor Repository (DCTD, National Cancer Institute). DCTD and ATCC employ short tandem repeat DNA fingerprinting, karyotyping, and cytochrome C oxidase I testing to authenticate cell lines. All cell lines were cultured for less than two months after thawing frozen stocks. U251 and HeLa were grown in DMEM supplemented with 10% fetal bovine serum (FBS). PANC-1 and PSN1 were grown in RPMI 1640 medium supplemented with 10% FBS. All cell lines were maintained at 37 °C in an atmosphere of 95% air/5% CO_2_. CX-5461 (obtained from DCTD) was dissolved in 50 mM NaH_2_PO_4_·H_2_O. Actinomycin D (ActD) and staurosporine (STS) were dissolved in dimethyl sulfoxide (DMSO). Cells were irradiated using a 320 kV X-ray source with a 2.0 mm aluminum filtration (300 kV peak, 10 mA) at a dose rate of 2 Gy/minute. Control cultures were mock irradiated.

### Clonogenic survival assay

Cells were seeded at clonal density in 6-well plates. 16 h after plating, cells were treated with CX-5461 or vehicle (50 mM NaH_2_PO_4_·H_2_O) for 1 h and then irradiated. The drug or vehicle was removed 24 h after irradiation. Ten to 14 days after plating, colonies were stained with 0.5% crystal violet in methanol. The number of colonies was determined, and the surviving fractions were calculated. Radiation survival curves were generated after normalizing to cytotoxicity induced by CX-5461. Data presented are the mean ± standard error of the mean (SEM) for three to four independent experiments. Statistical significance was calculated by Student’s t-test.

### qPCR

RNA was harvested from cells using TRIzol Reagent, according to the manufacturer’s protocol. Isolated RNA was treated with DNase to remove contaminating ribosomal DNA. RNA was reversed transcribed using the High Capacity RNA-to-cDNA Kit (Thermo Fisher Scientific). The resulting cDNA was used in qPCR reactions with Power SYBR Green PCR Master Mix (Thermo Fisher Scientific). Primer sequences are as follows: 45S F: 5′-ACCCACCCTCGGTGAGA-3′, 45S R: 5′-CAAGGCACGCCTCTCAGAT-3′, 7SK F: 5′-CCCCTGCTAGAACCTCCAAAC-3′, 7SK R: 5′-CACATGCAGCGCCTCATTT-3′. PCR reaction conditions were 50 °C for two minutes, 95 °C for 10 min, followed by 40 cycles of 95 °C for 15 s and 60 °C for one minute. Data were analyzed using Applied Biosystems 7500 System Software with the standard curve method. Data presented are the mean ± SEM for three independent experiments. Statistical significance was calculated by Student’s t-test.

### Apoptosis immunoblot analysis

Cells were washed with PBS, pelleted by centrifugation, and incubated in lysis buffer (150 mM NaCl, 50 mM Tris, pH 7.5; 0.3% Tween 20, 0.2% Triton X-100, 1X HALT protease inhibitor cocktail [Thermo Scientific], 1X phosphatase inhibitor cocktail 2 [Sigma Aldrich], and 1X phosphatase inhibitor cocktail 3 [Sigma Aldrich]) on ice for 10 min. The resulting lysates were cleared by centrifugation at 12,000 rpm for 5 min at 4 °C. The protein concentration of the cleared lysates was determined by BCA protein assay. Equal amounts of protein were separated on 4–20% SDS-PAGE gels, transferred to nitrocellulose membranes, and probed with primary antibodies. Membranes were cut prior to antibody hybridization (see Supplemental Figure). Antibodies used include PARP (Cell Signaling Technology #9542), caspase 3 (Cell Signaling Technology #9662), and α-tubulin (Cell Signaling Technology #2125). Proteins were visualized by HRP conjugated anti-rabbit IgG secondary antibody and incubation in ECL.

### Mitotic catastrophe

Cells grown in two-well chamber slides were fixed with 10% neutral buffered formalin, permeabilized with 0.2% Triton X-100 in PBS, and blocked with 5% goat serum, 1% bovine serum albumin (BSA), and 0.5% Tween-20 in PBS. The cytoplasm was stained by an overnight incubation with α-tubulin primary antibody (Cell Signaling Technology #2125) at 4 °C, and then a 2 h, room temperature incubation with goat anti-rabbit IgG Alexa Fluor 488 antibody. Coverslips were mounted using ProLong Gold Antifade Mountant with DAPI to stain nuclei. Slides were imaged on a Zeiss Axio Imager 2 with a 63× objective. Mitotic catastrophe was scored in 50–100 cells per treatment group. Cells were scored positive for mitotic catastrophe if they contained micronuclei, one nucleus with two or more lobes, or multiple nuclei^17^. Data presented are the mean ± SEM for three independent experiments. Statistical significance was calculated by Student’s t-test.

### γH2AX foci analysis

Cells grown in two-well chamber slides were fixed with 10% neutral buffered formalin, permeabilized with 0.2% Triton X-100 in PBS, and blocked with 5% goat serum, 1% bovine serum albumin, and 0.5% Tween-20 in PBS. Phosphorylated H2AX was detected by an overnight incubation with γH2AX primary antibody (Millipore Sigma #05-636) at 4 °C, and then a 2 h, room temperature incubation with goat anti-mouse IgG Alexa Fluor 488 antibody. Coverslips were mounted using ProLong Gold Antifade Mountant with DAPI to stain nuclei. Slides were imaged on a Zeiss Axio Imager 2 with a 63× oil immersion lens. γH2AX foci were counted in 50 cells per treatment group using Image J. Data presented are the mean ± SEM for three independent experiments. Statistical significance was calculated by Student’s t-test.

## Results

**Figure 1:**
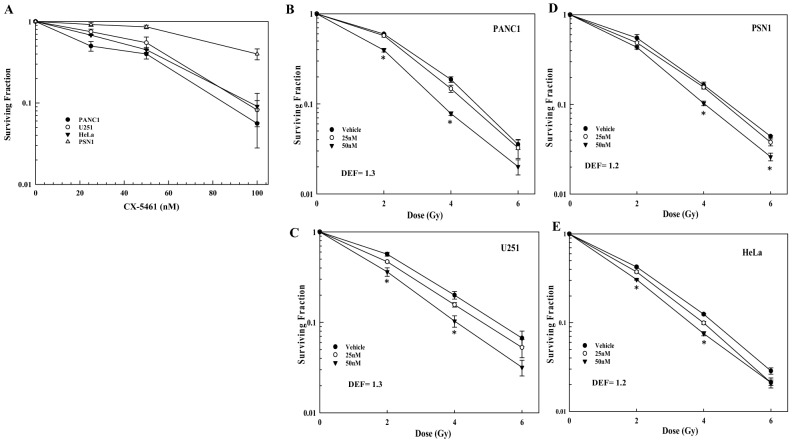
Tumor cell sensitivity to CX-5461 and the effects of CX-5461on radiosensitivity. **(A)** Tumor cell lines were treated with CX-5461 (25–100 nM); after 24 h drug containing media was removed and replaced with drug-free media, and colonies determined after 10–14 days. To determine the effects of CX-5461 on radiosensitivity **(B–E)** drug was added to cell culture media 1 h before irradiation. 24 h post-irradiation, drug containing media was removed and replaced with drug-free media, and colonies determined after 10–14 days. Dose enhancement factors (DEFs) were calculated at a surviving fraction of 0.1. Values represent the mean ± SEM for three to four independent experiments. * p < 0.05 by Student’s t-test.

In an initial analysis, clonogenic survival was used to determine the sensitivity to CX-5461 of 4 human tumor cell lines: PANC-1 and PSN1 (pancreatic adenocarcinoma), U251 (glioblastoma) and HeLa (cervical carcinoma). As shown in Fig. 1A, CX-5461 exposure (24 h) reduced survival in each cell line in a dose dependent manner, with PSN1 being the least sensitive. To evaluate its combination with radiation, we selected CX-5461 concentrations of 25 and 50 nM, at which survival remained sufficiently high in each of the cell lines to allow for the accurate evaluation of any potential radiosensitization. Specifically, cells were plated at clonal density, allowed to attach overnight, and CX-5461 (25 or 50 nM) added to culture media 1 h before irradiation. 24 h post-irradiation, cultures were rinsed, and fresh, drug-free media was added, with colonies determined 10–14 days later (Fig. 1B–E). The survival curves show that, while 25 nM had little to no effect on radiation-induced cell death, 50 nM CX-5461 significantly enhanced the radiosensitivity of each tumor cell line with dose enhancement factors (DEF) of 1.2–1.3, as determined at a surviving fraction of 0.1.

**Figure 2:**
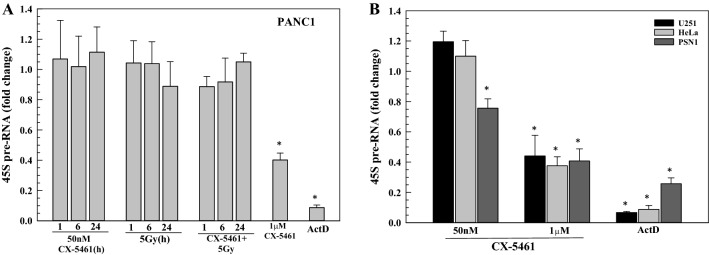
Effect of the radiosensitizing dose (50 nM) of CX-5461 on 45S rRNA transcription. **(A)** PANC1 cells were treated with 50 nM CX-5461 1 h prior to irradiation (5 Gy); RNA was harvested at the indicated times with 45S transcript levels determined by qPCR analysis and normalization to 7SK lncRNA. Treatment with 1 µM CX-5461 for 1 h and 20 ng/mL actinomycin D (ActD) for 2 h were used as positive controls for inhibition of 45S transcription. **(B)** U251, HeLa, and PSN1 cells were treated with 50 nM CX-5461 (24 h), 1 µM CX-5461 (1 h) or 20 ng/mL ActD (2 h). 45S transcript levels were then determined by qPCR analysis with normalization to 7SK lncRNA. Values represent the mean ± SEM for three independent experiments. * p < 0.05 by Student’s t-test.

The role of Pol I inhibition in CX-5461-induced radiosensitization was assessed using qPCR to quantitate 45S pre-rRNA transcript levels. Initial experiments were performed using PANC-1 cells with 45S transcript levels measured after exposure to 50 nM CX-5461, 5 Gy, or the combination (Fig. 2A). The radiosensitizing concentration of CX-5461 had no effect on 45S transcript levels out to 24 h of exposure, which was the length of treatment for the clonogenic survival assays. Radiation alone or the combination of radiation and 50 nM CX-5461 also had no effects on 45S transcripts. However, a significant reduction in 45S transcript level was detected after treatment of PANC-1 cells with 1 µM CX-5461, a concentration previously reported to inhibit Pol I and reduce 45S transcripts^8^, but too toxic to evaluate radiosensitization in clonogenic survival assays (see Fig. 1A). Actinomycin D (ActD, 20 ng/mL), which inhibits Pol I activity^18^, served as a positive control. The effects of the radiosensitizing concentration of CX-5461 (50 nM, 24 h) on 45S rRNA in the other tumor cell lines is shown in Fig. 2B. Although 50 nM CX-5461 induced a slight decrease (24%) in PSN1 cells, no significant change in 45S transcripts was detected in U251 or Hela cells, as observed in PANC-1. These data indicate that CX-5461 induced radiosensitization does not involve inhibition of Pol I.

**Figure 3:**
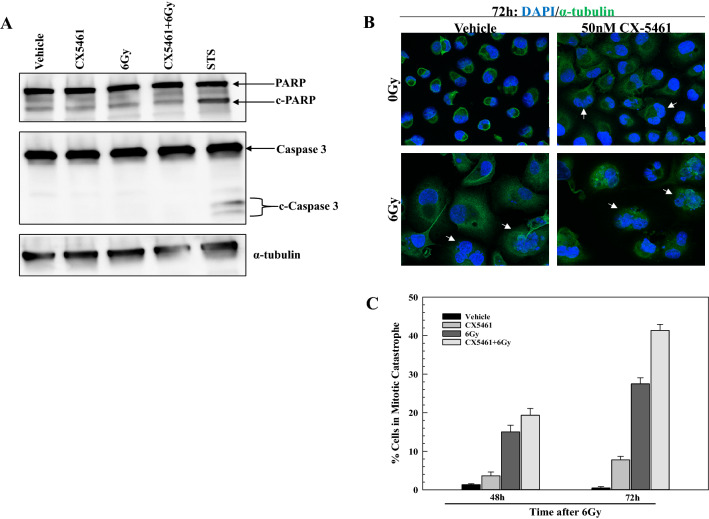
The effect of CX-5461, radiation and the combination on the mode of cell death in PANC-1 cells. (**A**) Western blot analysis of apoptosis in PANC-1 cells. 50 nM CX-5461 was added to cultures 1 h prior to irradiation (6 Gy), and cells were harvested at the indicated time points. Staurosporine (STS; 1 µM for 3 h) was used as positive control for apoptosis induction. Cell lysates were immunoblotted for PARP and caspase 3, with α-tubulin as a loading control. Full-length blots are presented in Supplementary Information. (**B**) Representative images of mitotic catastrophe in PANC-1 cells. 50 nM CX-5461 was added to cultures 1 h prior to irradiation (6 Gy) and removed 24 h post-irradiation with cells collected at 72 h. Examples of cells undergoing mitotic catastrophe are indicated by white arrows. (**C**) Quantitation of mitotic catastrophe. CX-5461 (50 nM) was added to cultures 1 h prior to irradiation (6 Gy) and removed 24 h post-irradiation with cells collected at the indicated time points. Cells containing micronuclei, a nucleus with 2 or more lobes, or multiple nuclei were scored positive for mitotic catastrophe. 50–100 cells were scored per treatment group. Values represent the mean ± SEM for three independent experiments. * p < 0.05 by Student’s t-test.

As an initial step towards understanding the mechanism through which CX-5461 enhances radiosensitivity, we focused on PANC-1 cells and the mode of cell death. Towards this end, we first defined the effect of the combination of CX-5461 and radiation on apoptotic cell death. CX-5461 has previously been reported to induce apoptosis in solid tumor cell lines at doses of 500 nM^19^ and 250 nM^20^ and to enhance the level of apoptosis induced by APR-246, a p53 activator, and INK128, an mTOR inhibitor^19,20^. Apoptosis was evaluated in PANC-1 cells at 24 h after exposure to 6 Gy with and without a 1 h pretreatment with the radiosensitizing dose of CX-5461 (50 nM). As shown in Fig. 3A, radiation had no effect on apoptosis as determined by PARP cleavage and caspase 3 cleavage. 50 nM CX-5461 alone did not induced detectable apoptosis; the combination of CX-5461 and radiation had no effect on apoptosis compared to vehicle treated cells. These data indicate the radiosensitization induced by CX-5461 does not involve modifications in apoptotic death.

In solid tumor cells, the dominant mode of radiation-induced cell death is through mitotic catastrophe. To evaluate whether CX-5461 induced radiosensitization involves this form of cell death, PANC-1 cells were treated with CX-5461, radiation, or the combination, and the percentage of cells in mitotic catastrophe was determined. Specifically, CX-5461 (50 nM) was added to culture media 1 h before irradiation (6 Gy); 24 h post-irradiation, cultures were rinsed and fresh, drug-free media was added with mitotic catastrophe evaluated at 48 and 72 h after irradiation. Representative images of mitotic catastrophe in PANC-1 cells are shown in Fig. 3B with quantitation presented in Fig. 3C. CX-5461 alone resulted in a slight, but significant increase in mitotic catastrophe at 72 h. Radiation alone increased mitotic catastrophe at 48 h with a further increase at 72 h. The combination of CX-5461 and radiation produced a significant increase in mitotic catastrophe as compared to radiation alone at 72 h. These data indicate that CX-5461 induced radiosensitization involves an increase in cells undergoing mitotic catastrophe, which suggests an effect on DNA double strand breaks (DSBs), the critical lesion responsible for radiation-induced cell death.

**Figure 4:**
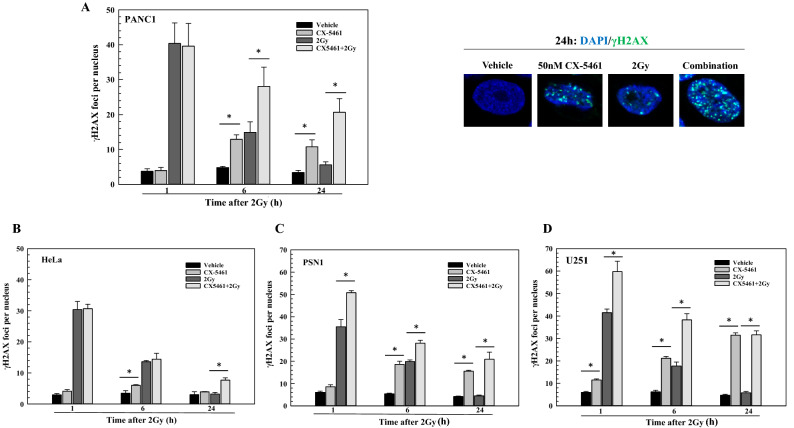
γH2AX foci analysis after cell exposure to CX-5461, 2 Gy, or the combination. γH2AX foci were determined in PANC-1 (**A**), HeLa (**B**), PSN1 (**C**), and U251 (**D**) cells. 50 nM CX-5461 was added to cultures 1 h prior to irradiation (2 Gy) and cells were collected at the indicated time points for foci analysis. γH2AX foci were counted in 50 cells per treatment group. Representative images of γH2AX foci in PANC-1 at 24 h are shown in (**A**). Values represent the mean ± SEM for three independent experiments. * p < 0.05 by Student’s t-test.

Accordingly, to further investigate the mechanism of CX-5461-induced radiosensitization, γH2AX foci analysis was used to evaluate DNA DSBs in each of the 4 cell lines shown in Fig. 1B. γH2AX foci induced after irradiation correspond to the number of DSBs generated, whereas γH2AX foci dispersal correlates with DSB repair^21,22^. Following the same protocol used in the clonogenic survival experiments, CX-5461 (50 nM) was added to culture media 1 h before irradiation (2 Gy) and γH2AX nuclear foci determined at times out to 24 h. Irradiation of PANC-1 cells resulted in the standard response in that there was an initial increase in γH2AX foci at 1 h followed by a reduction to control levels by 24 h (Fig. 4A). While CX-5461 treatment alone had no effect on the number of γH2AX foci at the 1 h time point, after 6 and 24 h of exposure, significant increases were detected. No difference in foci levels were apparent between 2 Gy and the CX-5461/2 Gy combination at 1 h after irradiation, suggesting that CX-5461 has no effect on the initial levels of radiation-induced DSBs. However, at 6 and 24 h after irradiation, the number of γH2AX foci remaining was significantly greater in the cells treated with the CX-5461/radiation combination than in cells exposed to 2 Gy only. In HeLa cells (Fig. 4B), CX-5461 treatment alone resulted in a slight but significant increase in γH2AX foci at 6 h, which returned to control levels at 24 h. For cells receiving the combination treatment, the number of γH2AX foci remaining at 24 h was significantly above the number of foci remaining at 24 h in cells treated with radiation alone. Thus, in PANC-1 and HeLa cells, the persistence of γH2AX foci in irradiated cells that were treated with CX-5461 suggests an inhibition of DNA DSB repair.

The same experiment was performed using PSN1 and U251 cells. In PSN1 cells (Fig. 4C) CX-5461 alone had no effect on the number of γH2AX foci at 1 h; at 6 h there was a significant increase that was maintained out to 24 h. At 1 h the number of γH2AX foci in cells receiving the CX-5461/2 Gy combination was greater than the number in cells treated with 2 Gy alone, suggesting an increase in the initial level of radiation-induced DSBs. For cells receiving the combination treatment, the number of γH2AX foci remaining at 24 h was significantly above radiation alone, consistent with an inhibition of DSB repair. As shown in Fig. 4D, in U251 cells CX-5461 alone increased foci numbers at 1 h, which were then further increased at 6 and 24 h. At 1 h the number of γH2AX foci in cells receiving the CX-5461/2 Gy combination was greater than the number in cells treated with 2 Gy alone, again suggesting an increase in the initial level of radiation-induced DSBs. At 24 h, the number of foci remaining in CX-5461/2 Gy combination was increased compared to the number of foci remaining in 2 Gy alone. However, because the number of foci remaining in the combination group was similar to that detected in CX-5461 alone, the effects of CX-5461 on the repair of radiation-induced DSBs is unclear. The data in Fig. 4 suggest that CX-5461 inhibits the repair of radiation-induced DSBs and/or increases the initial level of radiation-induced DSBs in a cell type specific manner.

## Discussion

Based on its ability to inhibit Pol I and reduce rDNA transcription, CX-5461 is under evaluation as a treatment for hematological and solid malignancies (NCT02719977, NCT04890613, and ^7^). Because ribosome biogenesis has also been implicated as a determinant of tumor cell radiosensitivity, the aim of the studies presented here was to determine whether CX-5461 may act as a radiosensitizing agent. As shown here, CX-5461 enhanced the radiosensitivity of 4 cell lines generated from 3 different solid tumor types typically treated with radiotherapy. A previous study^14^ suggested that CX-5461 enhances radiation-induced cell killing. However, in that study cell death was determined using a 4-day proliferation assay, which does not provide an accurate measure of radiosensitivity^15^, and the effects of radiation alone were not shown, which makes radiosensitization difficult to assess. In the study shown here, radiosensitization of tumor cell lines by CX-5461 was established using clonogenic assays, the gold standard for determining radiosensitivity as well as its modification.

Our initial hypothesis was that the increased radiosensitivity detected in CX-5461 treated cells was causally related to the reduction in rDNA transcription. However, at the radiosensitizing concentration of CX-5461 (50 nM), no consistent reduction in 45S transcripts was detected. Adding the additional stress of radiation also had no effect 45S levels, as shown by the CX-5461 + 5 Gy combination treatment (Fig. 2A). In contrast, a significant loss of 45S was detected after treatment with 1 µM CX-5461, which is in the range previously reported to reduce rDNA transcription in solid tumor cell lines^8,14^. The data presented thus indicate that whereas CX-5461 induces radiosensitization, this effect cannot be attributed to a reduction in ribosome biogenesis. Whereas Ismael et al., reported a high degree of synergy between low doses of CX-5461 (6.25–50 nM) and radiation, Pol I activity was only determined at 500–1000 nM CX-5461^14^, which questions the role of Pol I inhibition in the reported synergistic effects.

With respect to the mechanism mediating the CX-5461 induced radiosensitization, the initial finding of an increase in cells undergoing mitotic catastrophe suggested a role for the DNA damage response. CX-5461 has been shown to activate the DNA damage response kinases ATM and ATR^23–25^. We focused on DSBs, the lesions responsible for radiation-induced cell death, which were evaluated according to γH2AX foci. It has been previously reported that CX-5461 alone induces γH2AX in solid tumor cell lines after exposure to doses in the range of 100 nM to 1 µM^16,25,26^. This was attributed to multiple, not necessarily mutually exclusive effects, including CX-5461 mediated replication fork collapse^25^, G4 stabilization^16^, and topoisomerase poisoning^26^. CX-5461 binding to and stabilization of G4 results in transcription-dependent topoisomerase poisoning^27^ and replication-dependent DNA damage^16,28^, which can include replication fork collapse. Indeed, CX-5461 is structurally related to CX-3543 (quarfloxin), a known G4 ligand and a derivative of the fluoroquinolone family, a class of compounds that includes antibiotics targeting bacterial topoisomerases^29^. As shown here, exposure to the radiosensitizing dose of CX-5461 alone resulted in a range of γH2AX foci induction from approximately 6 foci per nucleus in HeLa cells to 30 foci per nucleus in U251 cells. This would appear consistent with the cell type variability in the frequency of G4 structures^30^. Comparison of the survival curves shown in Fig. 1A and γH2AX foci induced does not suggest a causal relationship between the CX-5461 induced DSBs and the reduction in cell survival. Thus, additional mechanisms may mediate toxicity from CX-5461 treatment.

CX-5461 delayed the dispersal of radiation-induced γH2AX foci in PANC1, HeLa and PSN1, indicative of an inhibition of the repair of radiation-induced DSBs and consistent with radiosensitization. Another G4 stabilizing agent RHPS4 was shown to enhance the radiosensitivity of glioblastoma cell lines and inhibit DSB repair as measured by γH2AX foci^31^. However, in PSN1 and U251 cells CX-5461 also increased the initial level of radiation-induced DSBs, which is consistent with G4 structures being enriched at sites of DNaseI hypersensitivity and low nucleosome occupancy^32,33^. Given that G4 structures play multiple roles in regulating DNA structure and metabolism^34^, CX-5461 may also increase radiosensitivity through more than one mechanism. Regardless of the γH2AX response, all cell lines receiving the combination of radiation and CX-5461 exhibited unresolved γH2AX foci at 24 h post-irradiation, which is typically thought to lead to radiation-induced cell death^35^.

With respect to cancer treatment, the data presented here suggest that CX-5461 may be of value in combination with radiotherapy. However, the radiosensitization detected herein occurs at a dose significantly less than that required to inhibit rDNA transcription and is consistent with targeting of G4 structures. Understanding the target for CX-5461-induced radiosensitization should be of value in the potential application of the combination of CX-5461 and radiotherapy.

## Supplementary Information

Supplementary Information.

## Data Availability

All data generated or analyzed during this study are included in this published article.
