# Supplementary material for: CX-5461 induces radiosensitization through modification of the DNA damage response and not inhibition of RNA polymerase I
Source: Sci Rep. 2022 Mar 8;12:4059. doi: 10.1038/s41598-022-07928-4 (PMC8904802; doi:10.1038/s41598-022-07928-4)
Supplement: Supplementary file 1 — Supplementary Information. [file 41598_2022_7928_MOESM1_ESM.pdf]

Supplementary Information

Uncropped western blot for Figure 3

Title: CX-5461 induces radiosensitization through modification of the DNA damage response and not inhibition of RNA polymerase I

Authors: Stacey L. Lehman, Kayla R. Schwartz, Shrankhla Maheshwari, Kevin Camphausen, Philip J. Tofilon

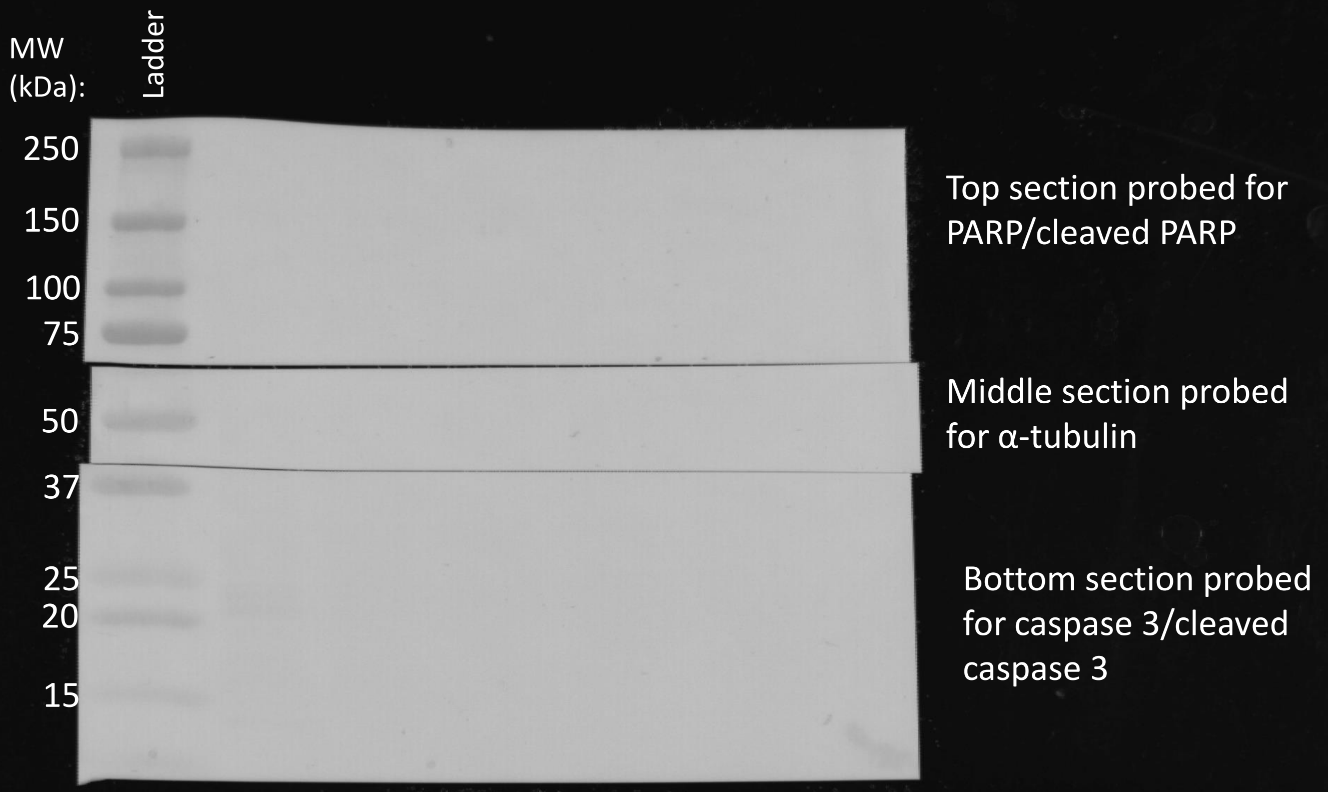

Note: Membrane cut into separate pieces prior to hybridization. The top of the blot corresponds to the top of the membrane.

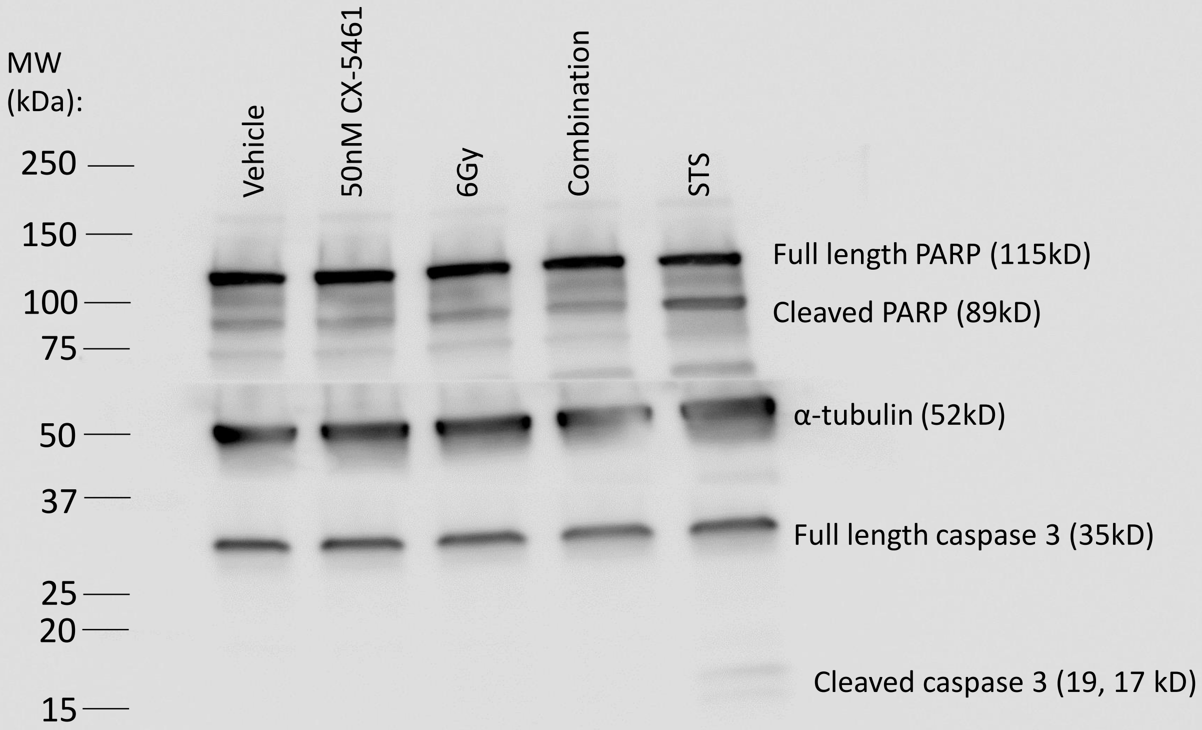

Used PARP and tubulin from this exposure in Figure 3.

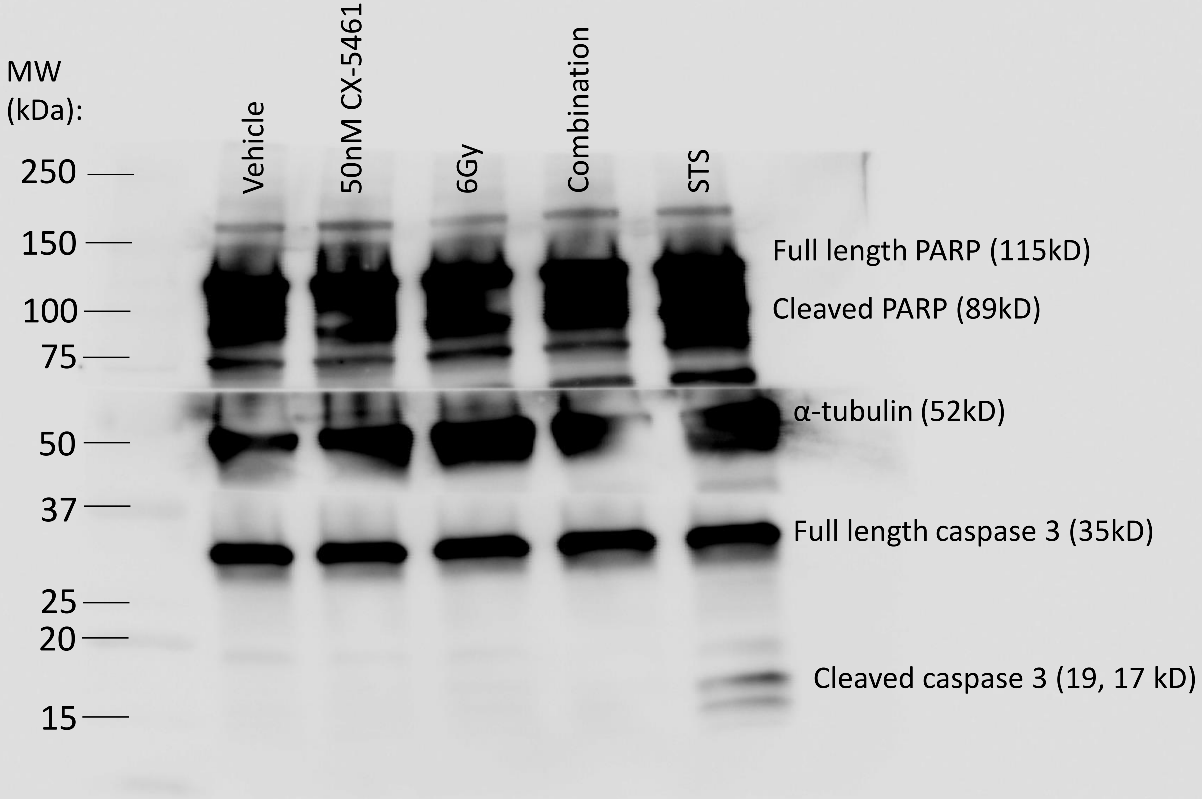

Used caspase 3 from this exposure in
Figure 3.

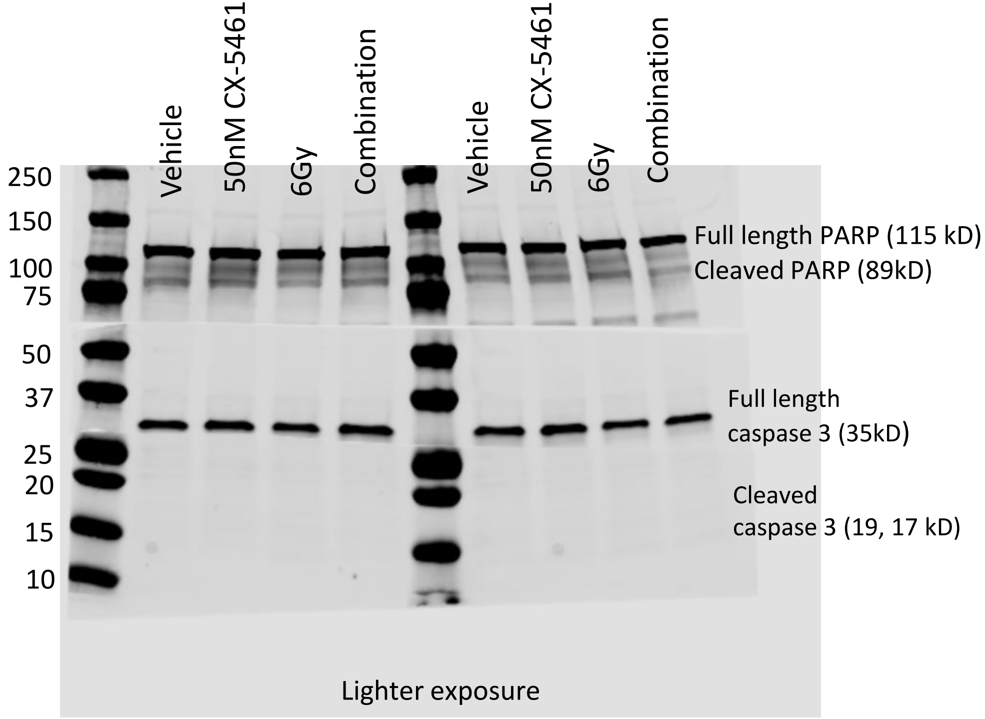

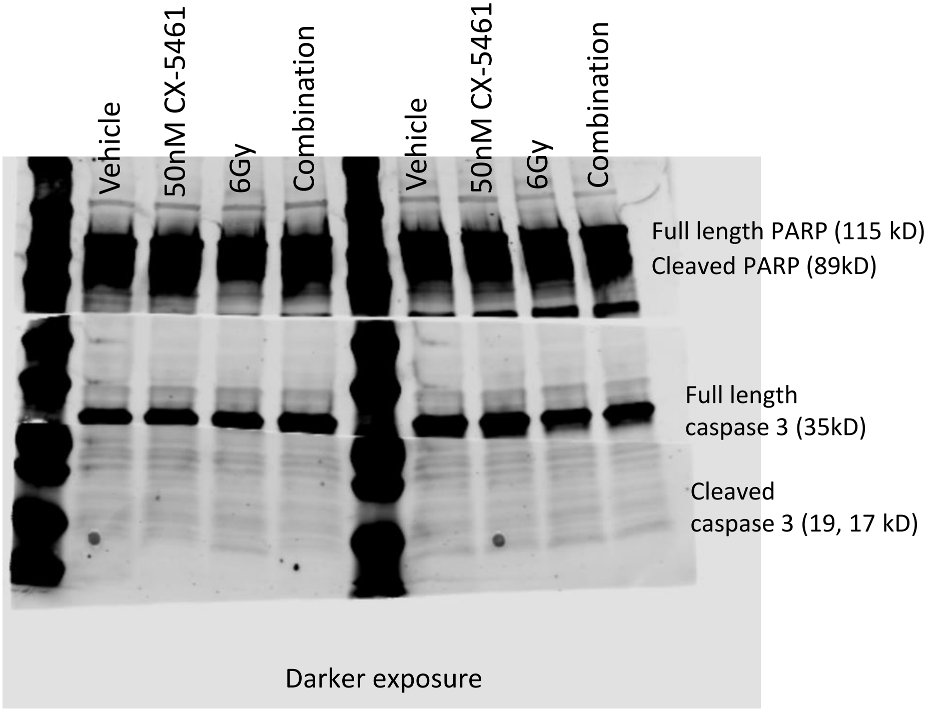

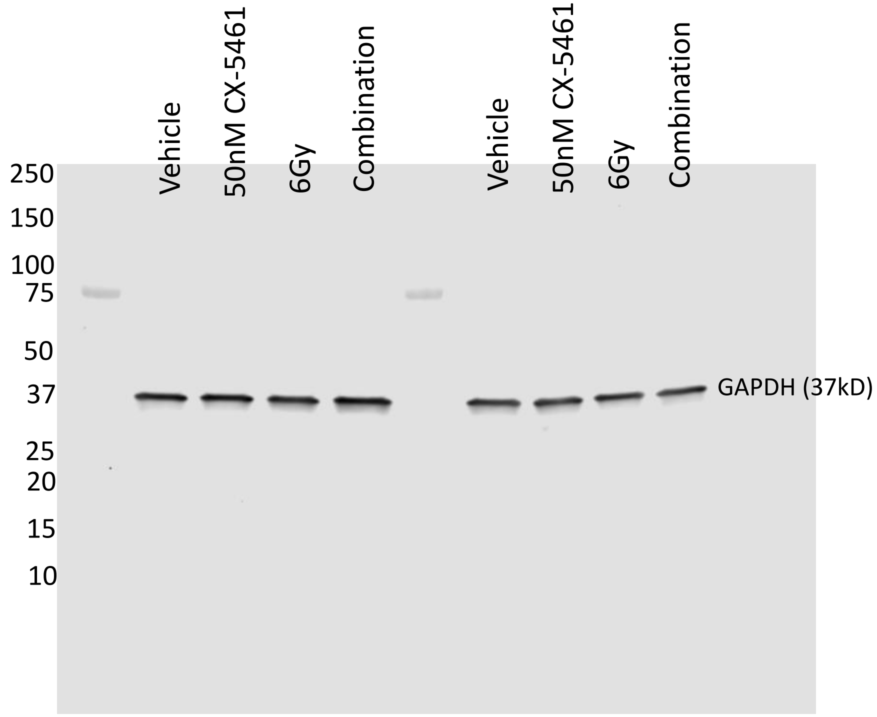

Two additional replicates of western blot experiment. Lysates were harvested from two biologically independent experiments and run on the same gel. The membrane was cut into separate pieces prior to hybridization. The top of the blot corresponds to the top of the membrane.

Primary antibodies:
PARP: Cell Signaling Technology #9542
Caspase 3: Cell Signaling Technology #9662
Cleaved Caspase 3: Cell Signaling Technology #9661
GAPDH: Thermo Fisher #437000

PARP, caspase 3, and cleaved caspase 3 were detected with α-rabbit IRDye 680RD secondary antibody, and GAPDH was detected with α-mouse IRDye 800CW secondary antibody. Blot images were acquired on a Licor Odyssey CLx.
